# Tirapazamine sensitises hypoxic homologous recombination-proficient NSCLC to PARP inhibition

**DOI:** 10.1038/s41420-026-03233-5

**Published:** 2026-07-02

**Authors:** Yanyan Jiang, Nuria Vilaplana Lopera, Hala Estephan, Yizhou Wang, Ftoon Aljarbou, Ester M. Hammond, Amato J. Giaccia, Eui Jung Moon

**Affiliations:** 1 Department of Oncology, University of Oxford, Oxford, UK; 2 Department of Psychiatry, University of Oxford, Oxford, UK

**Keywords:** Cancer microenvironment, Cancer therapy

## Abstract

PARP inhibitors (PARPi) are clinically effective in homologous recombination (HR)-deficient tumours, but their application in HR-proficient cancers remains limited. Although hypoxia suppresses HR, this repression alone does not adequately sensitise cancer cells to PARPi due to insufficient endogenous DNA damage. We hypothesised that introducing a hypoxia-selective source of DNA damage could enhance PARPi efficacy. Here, we show that the hypoxia-activated prodrug tirapazamine (TPZ) synergises with the PARPi talazoparib to induce cytotoxicity in HR-proficient non-small cell lung cancer (NSCLC) cells, with preferential activity under hypoxia. Mechanistically, this synergy is driven by increased DNA double-strand breaks (DSBs) and replication stress. These lesions are largely resolved in normoxia but persist in hypoxia. In hypoxic spheroids derived from HR-proficient NSCLC cell lines, the TPZ and talazoparib combination significantly inhibited spheroid growth. In subcutaneous xenografts, the combination similarly suppressed tumour growth in the highly hypoxic A549 and CORL105 models, while showing minimal efficacy in the less hypoxic Calu-3 and H3122 tumours. This study highlights the potential of hypoxia-targeted DNA-damaging agents to expand PARPi utility to HR-proficient cancers and demonstrates that hypoxia enhances the therapeutic effect of this combination.

## Introduction

PARP inhibitors (PARPi) inhibit the catalytic activity of Poly-(ADP-ribose) Polymerase (PARP), especially PARP1, a nuclear enzyme essential for repairing DNA single-strand breaks (SSBs) via base excision repair (BER) and modulating double-strand break (DSBs) repair [1, 2]. Beyond enzymatic inhibition, PARPi also trap PARP1 at DNA lesions, causing replication fork collapse. Unrepaired SSBs are then converted into lethal DSBs during replication [3, 4]. Since eukaryotic cells predominantly rely on homologous recombination (HR) for DSB repair, PARPi selectively induce synthetic lethality in HR-deficient cells [5, 6]. To date, four PARPi - olaparib, rucaparib, niraparib and talazoparib - have been clinically approved as monotherapies for HR-deficient cancers, including breast, ovarian, pancreatic, and prostate [7].

Although PARPis have shown marked efficacy in HR-deficient cancers, their activity in HR-proficient tumours remains limited. Extending PARPi benefit to HR-proficient cancers could have substantial clinical impact. A promising approach is to exploit contextual synthetic lethality, where hypoxia-driven reductions in HR gene expression and function render cells more sensitive to PARPi under severe hypoxia (≤ 0.2% O_2_) [8]. This concept, proposed nearly 15 years ago, has spurred extensive preclinical and clinical research to expand the use of PARPi beyond HR-deficient settings, particularly through combination therapies [8–11].

Hypoxia is a hallmark of most solid tumours, both in human cancers and animal tumour models [12, 13]. Although hypoxia can impair HR proficiency in cancer cells [8, 14, 15], robust single-agent activity of PARPi has not been consistently observed under hypoxic conditions. Indeed, we recently showed that mild hypoxia (2% O_2_) can confer resistance to PARPi even in HR-deficient tumours [16]. Consistent with this, PARPi-resistant breast tumours with germline BRCA1/2 mutations exhibited a hypoxic and/or stemness-associated gene expression signature [17]. These findings suggest that while hypoxia suppresses HR, this alone may be insufficient to sensitise cancer cells to PARPi due to low levels of endogenous DNA damage. We therefore hypothesised that an additional source of DNA damage, selectively targeting hypoxic cells, may be required to fully exploit this vulnerability. To test this hypothesis, we combined PARPi with tirapazamine (TPZ), a well-characterised hypoxia-activated prodrug that induces DNA damage selectively in hypoxic cells [18]. Importantly, TPZ predominantly induces SSBs over DSBs, thereby increasing reliance on PARP-dependent BER for DNA damage repair [19].

In this study, we investigated whether TPZ could sensitise HR-proficient NSCLC cells to PARPi and explored the underlying mechanisms. Using a panel of HR-proficient NSCLC cell lines, we examined the effects of TPZ and talazoparib on cytotoxicity, DNA damage response, and replication stress under both normoxic and hypoxic conditions. Furthermore, we assessed the therapeutic efficacy of this combination in spheroids and subcutaneous xenograft models with varying hypoxia levels. Our findings reveal a therapeutic opportunity to extend PARPi use beyond HR-deficient cancers, with combination therapy using a hypoxia-targeting agent further enhancing this potential.

## Results

### Talazoparib and TPZ synergistically reduce survival in HR-proficient NSCLC cells, preferentially under hypoxia

**Fig. 1:**
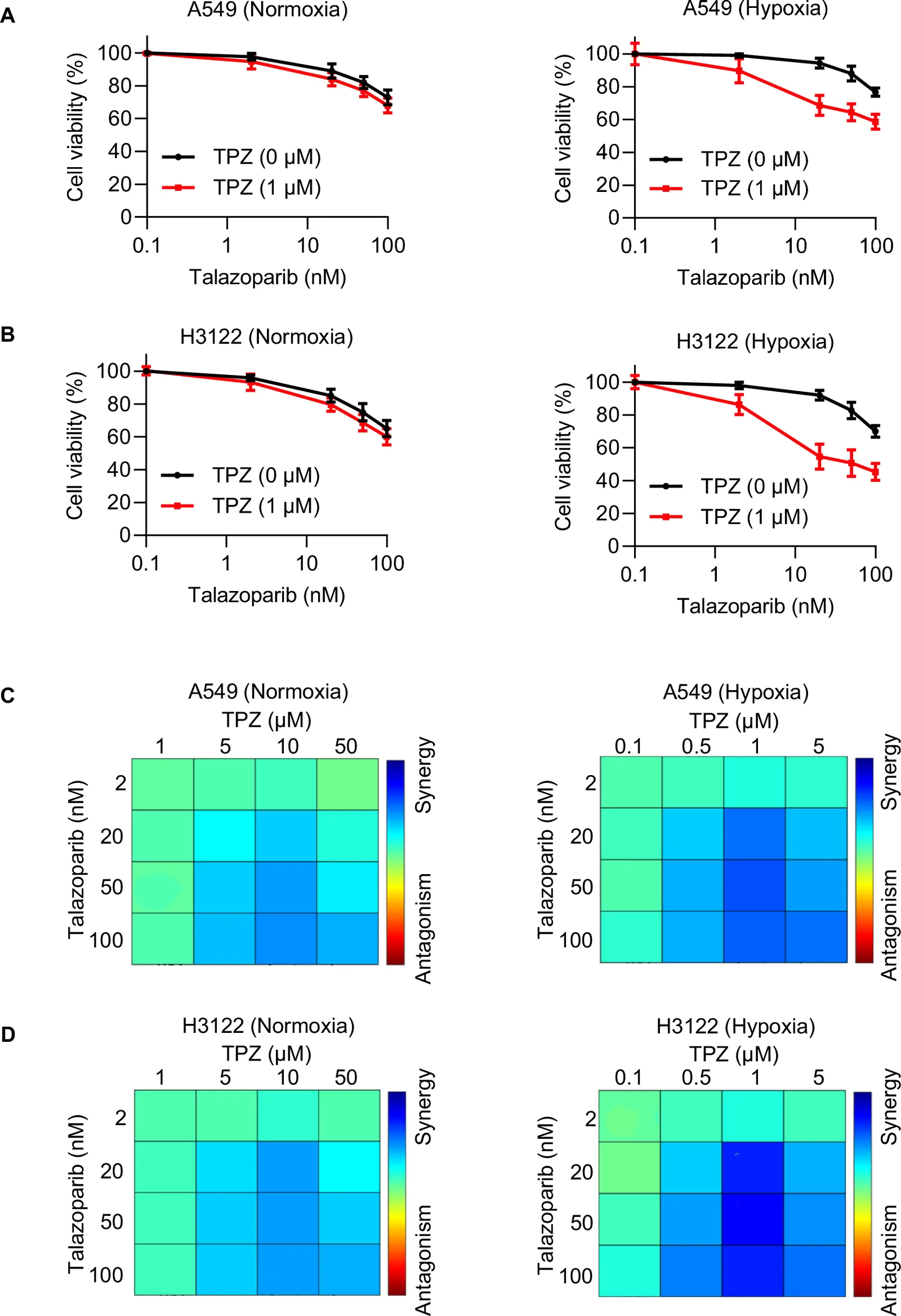
Talazoparib and TPZ synergistically reduce the survival of HR-proficient NSCLC cells, preferentially under hypoxia. **A**, **B** A549 and H3122 cells were treated with talazoparib (0.1–100 nM) and TPZ (0 or 1 µM) for 4 days under normoxia or hypoxia. Cell viability, which was assessed by CCK-8 assays, showed that TPZ sensitised A549 (**A**) and H3122 (**B**) cells to talazoparib only under hypoxia. The x-axis is shown on a log₁₀ scale. Data are presented as means ± SD (*n* = 3). **C**, **D** The highest single agent (HSA) synergy analysis of talazoparib and TPZ in A549 (**C**) and H3122 cells (**D**). Cells were treated with the indicated doses of talazoparib and TPZ for 4 days under normoxic or hypoxic conditions. Cell viability was then assessed by the CCK-8 assay and drug interactions were analysed based on the viability data (*n* = 3).

HR proficiency of the NSCLC cell lines used in this study (A549, H3122, CORL105, and Calu-3) was validated by assessing RAD51 foci formation following ionising radiation (Fig. S1). Consistent with previous reports in A549 and H3122 cells [20], RAD51 foci formation was significantly increased 4 h after 6 Gy irradiation. This was further confirmed using DLD1 parental and BRCA2-deleted cells [21] as HR-proficient and HR-deficient controls, respectively. To assess whether TPZ sensitises HR-proficient NSCLC cells to PARPi, A549 and H3122 cells were treated with varying concentrations of talazoparib with or without TPZ (1 µM) under either normoxic (21% O_2_) or hypoxic (1% O_2_) conditions. The drugs were applied for 4 days to capture potential dose-dependent interactions between the two agents. The combination of talazoparib with TPZ significantly reduced cell viability under hypoxia compared with talazoparib alone, whereas under normoxia neither talazoparib nor its combination with TPZ achieved comparable cytotoxicity (Fig. 1A, B). Using the combination benefit (combenefit) analysis software, which quantitatively evaluates drug interactions, we further confirmed a stronger synergistic effect under hypoxia (Fig. 1C-D). For example, 20 nM talazoparib combined with 1 μM TPZ produced 5.4- and 5.8-fold higher synergy scores in A549 and H3122 cells, respectively, under hypoxia compared with normoxia. Based on these findings, subsequent experiments were conducted using these treatment conditions.

### Talazoparib and TPZ combination enhance DNA damage in HR-proficient NSCLC cells

We hypothesised that TPZ-induced DNA damage under hypoxia would sensitise cells to PARP inhibition. To test our hypothesis, A549 and H3122 cells were treated with talazoparib, TPZ alone or their combination. DNA damage was assessed by alkaline comet assays for SSBs and neutral comet assays for DSBs.

**Fig. 2:**
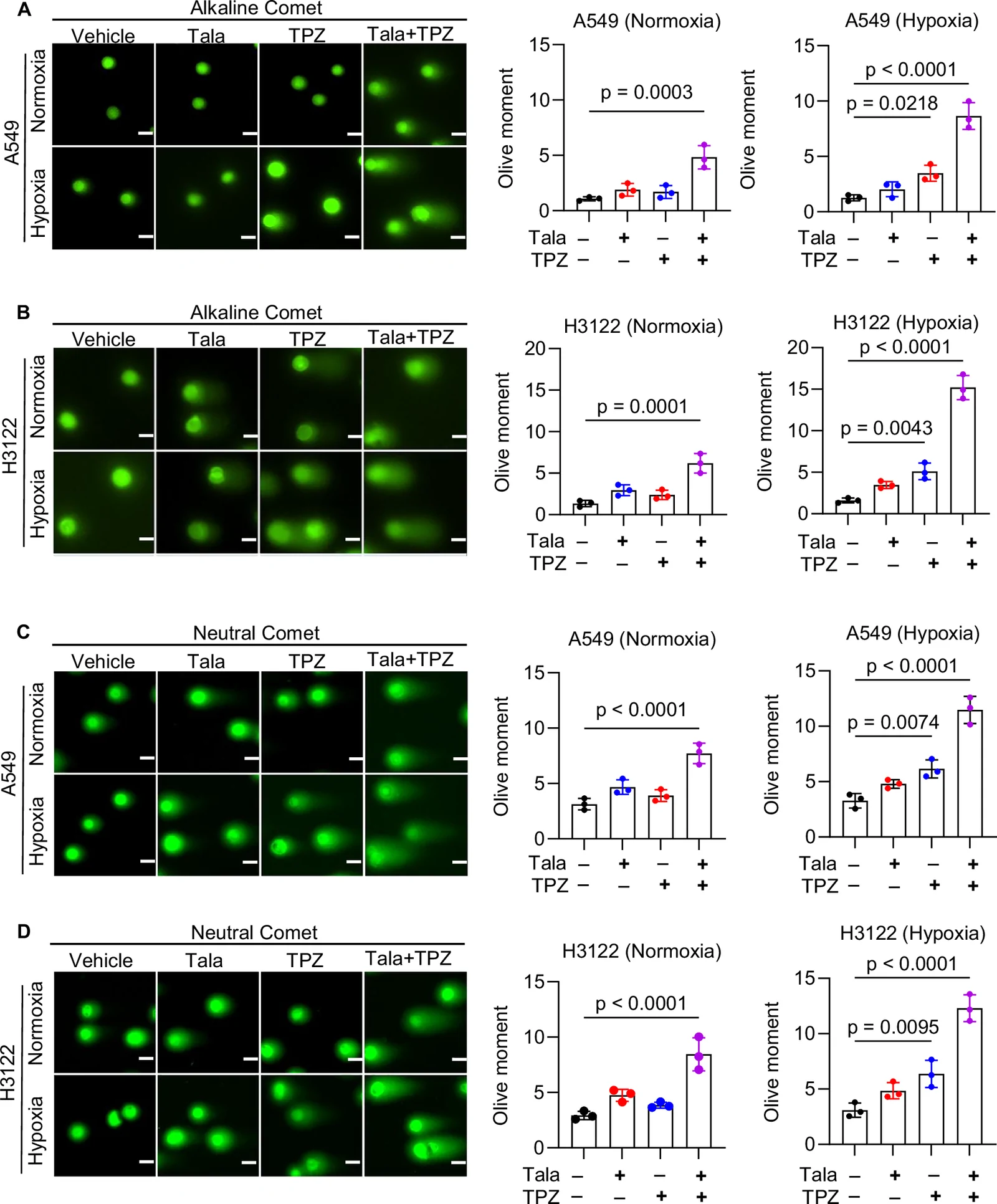
Talazoparib and TPZ combination enhance DNA damage in HR-proficient NSCLC cells. A549 and H3122 cells were treated with vehicle, talazoparib (20 nM), TPZ (1 µM), or talazoparib + TPZ for 24 h under normoxic or hypoxic conditions. **A**, **B** DNA SSBs were assessed using the alkaline comet assay in A549 (**A**) and H3122 (**B**). While TPZ increased SSBs only under hypoxia, the combination of talazoparib and TPZ enhanced SSB formation, with the most pronounced increase occurring specifically under hypoxic conditions. **C**, **D** DNA DSBs were evaluated using neutral comet assay. Consistent with the SSB data, DSBs measured by neutral comet assays were significantly increased by TPZ only under hypoxia, and the combination produced an even greater induction, particularly in hypoxic conditions. Data are presented as means ± SD (*n* = 3). Statistical significance was determined by one-way ANOVA with Dunnett’s multiple comparisons. Tala: Talazoparib, Scale bars: 25 μm.

Alkaline comet assays showed that TPZ alone significantly increased comet tail lengths in hypoxia but not in normoxia. Interestingly, the combination treatment significantly increased comet tail lengths under both normoxia and hypoxia, but the effect was substantially stronger under hypoxia (Fig. 2A, B). Similarly, neutral comet assays showed that TPZ alone significantly increased comet tail lengths in hypoxia, but not in normoxia (Fig. 2C, D). This effect was further enhanced by the combination treatment under both normoxia and hypoxia, again showing a more pronounced effect under hypoxia. Together, these findings indicate that the combination of TPZ and PARP inhibition induces greater DNA damage, particularly in hypoxic environments.

### Talazoparib and TPZ combination elevates reactive oxygen species (ROS)

**Fig. 3:**
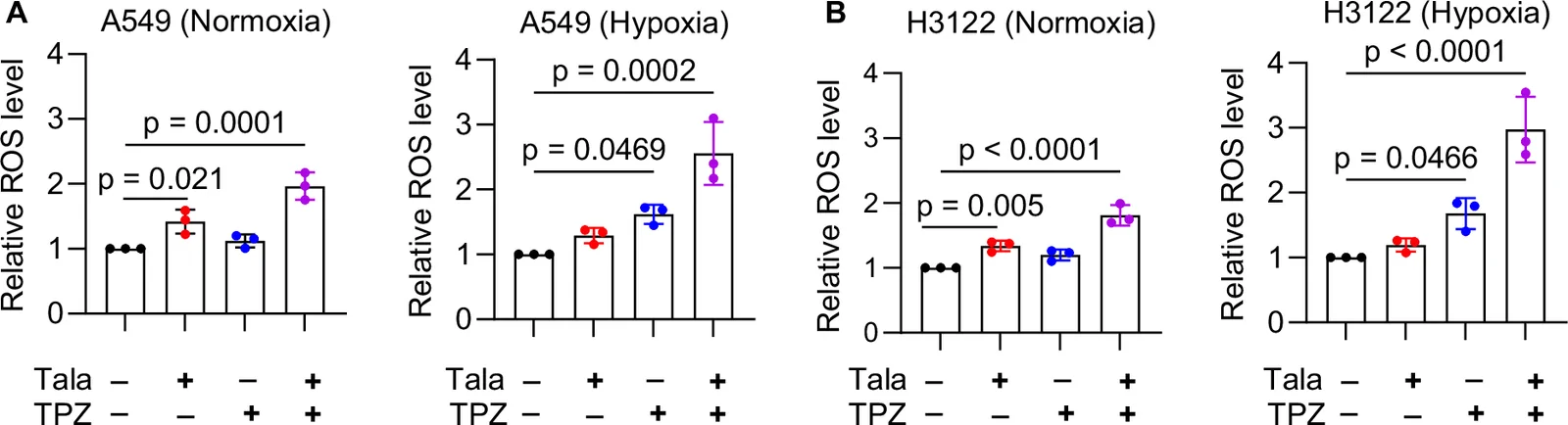
Talazoparib and TPZ combination elevates reactive oxygen species (ROS). **A**, **B** A549 and H3122 cells were treated for 24 h with talazoparib, TPZ, or in combination under normoxic or hypoxic conditions. Cellular ROS levels were measured by CellROX flow cytometry. Quantification of ROS levels in A549 (**A**) and H3122 (**B**) showed that talazoparib increased ROS production under normoxia whereas hypoxic conditions enhanced TPZ-induced ROS. Combination treatment further enhanced ROS particularly under hypoxia. Data are presented as means ± SD (*n* = 3). Statistical significance was determined by one-way ANOVA with Dunnett’s multiple comparisons.

TPZ is known to induce oxidative DNA damage predominantly under hypoxia [18], whereas PARP inhibition has been reported to increase intracellular ROS under normoxia [16, 22]. Consistent with these previous studies, we observed that talazoparib increased ROS levels only under normoxic conditions, while TPZ produced higher ROS specifically under hypoxia in both A549 and H3122 cells (Fig. 3A, B). Notably, the combination further increased ROS under both oxygen conditions, with the strongest effect observed in hypoxia. Together, these results suggest that TPZ and talazoparib cooperatively enhance oxidative stress, particularly in low-oxygen environments.

### Hypoxia impairs the resolution of DNA damage and replication stress

**Fig. 4:**
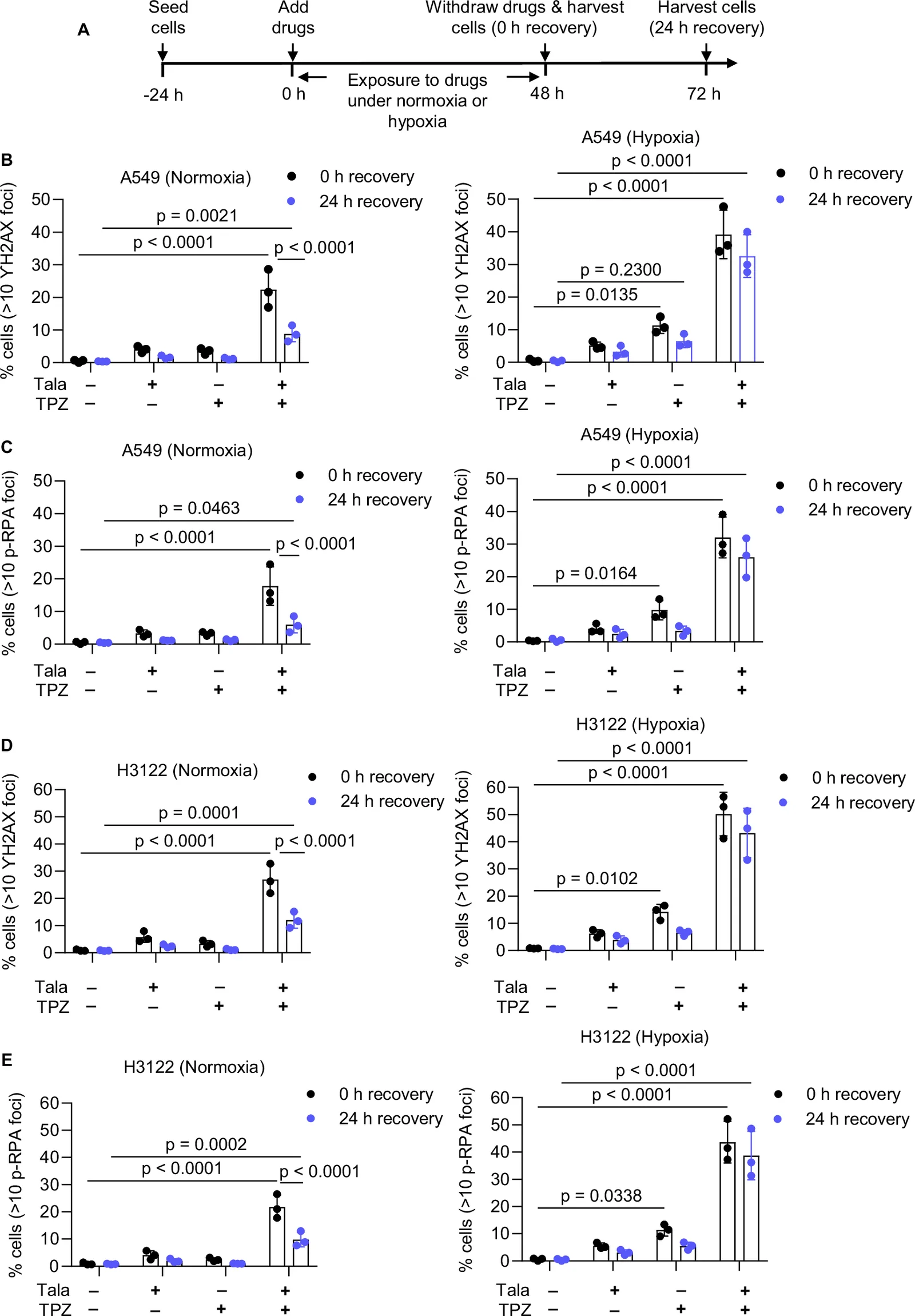
Hypoxia impairs the resolution of DNA damage and replication stress. **A** Schematic of DNA repair kinetics experiment. A549 and H3122 cells were plated and cultured overnight, and then treated for 48 h under normoxia or hypoxia with vehicle, talazoparib (20 nM), TPZ (1 µM), or talazoparib + TPZ. Following treatment, drug-containing medium was replaced with fresh drug-free medium under normoxia or hypoxia for cell recovery. Cells were collected at 0 or 24 h post-drug withdrawal for immunofluorescence staining of γH2AX (green)/p-RPA (red)/DAPI (blue). **B**–**E** A549 and H3122 cells with > 10 γH2AX foci (**B**, **D**) or > 10 p-RPA foci (**C**, **E**) were quantified. TPZ increased DNA damage and replication stress only under hypoxia, whereas the combination treatment elevated both under normoxia and hypoxia, with a substantially greater increase in hypoxia. After drug withdrawal, DNA damage and replication stress declined in normoxic cells, but persisted in hypoxic cells. Data are presented as means ± SD (*n* = 3). Statistical significance was determined by Two-way ANOVA with Sidak’s multiple comparisons.

While the combination of TPZ and talazoparib clearly generated more DNA damage under hypoxia, it was unclear whether hypoxia-associated defects in DNA repair contributed to this difference. Therefore, we evaluated DNA repair capacity by monitoring DNA damage and replication stress markers over a 24 h recovery period following drug withdrawal (Fig. 4A). A549 and H3122 cells were treated for 48 h under normoxic or hypoxic conditions with talazoparib, TPZ alone or their combination, followed by replacement with drug-free medium. γH2AX, a marker for DSBs and phospho-RPA (Ser33), which indicates replication stress were assessed by immunofluorescent staining immediately after drug withdrawal and 24 h later.

Consistent with the comet data shown previously (Fig. 2), 48 h of TPZ treatment significantly increased γH2AX- and phospho-RPA-positive cells only under hypoxia. Notably, the combination treatment produced an even stronger induction, again specifically under hypoxic conditions. However, 24 h after drug removal, γH2AX- and phospho-RPA-positive cells in the combination group significantly decreased under normoxia, but remained elevated under hypoxia, suggesting that hypoxia compromises the efficient repair of DNA damage and the resolution of replication stress (Fig. 4B–E; Fig. S2A–D). Given the persistent γH2AX and phospho-RPA signals under hypoxia (Fig. 4), we next examined HR by assessing RAD51 expression and foci formation. RAD51 protein levels were reduced following 48 h exposure to 1% O₂ alone (Fig. S3A), in line with previous reports showing that hypoxia downregulates RAD51 and HR activity [8, 14, 15]. Under normoxia, following 48 h of drug treatment, talazoparib increased RAD51 foci formation, and this effect was more pronounced when combined with TPZ. In contrast, talazoparib alone did not increase RAD51 foci under hypoxia (Fig. S3B). Interestingly, despite reduced RAD51 protein levels, TPZ induced a marked increase in RAD51 foci at 1% O₂, which was further enhanced by the combination and was higher than under normoxia. Together, these findings suggest that, even with reduced RAD51 expression, the high levels of DNA damage induced under hypoxia may drive increased RAD51 foci formation. This likely reflects the accumulation of DNA damage and replication stress beyond the capacity of HR to fully resolve.

### Combination of talazoparib and TPZ inhibits growth of hypoxic HR-proficient NSCLC spheroids

**Fig. 5:**
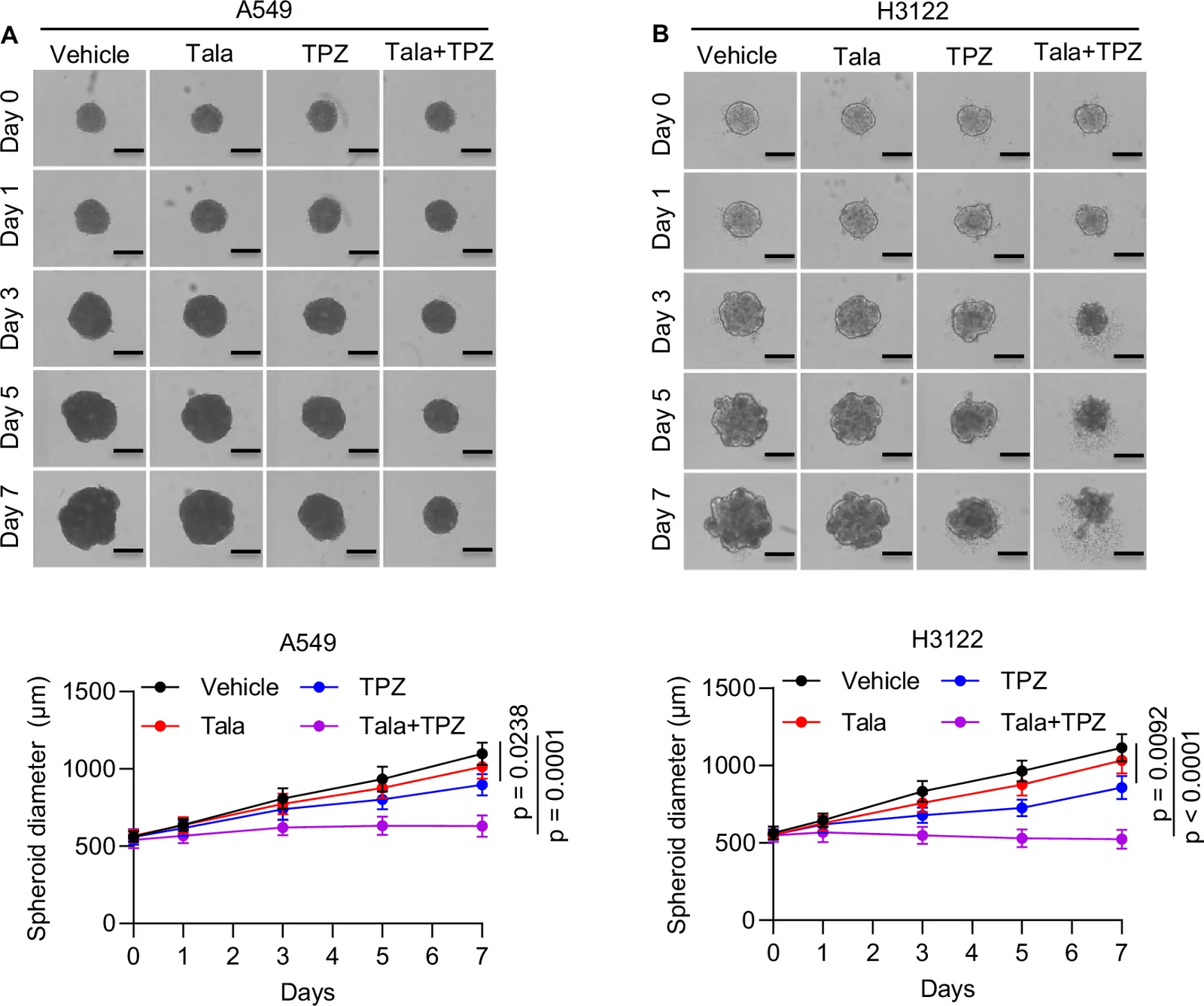
Combination of talazoparib and TPZ inhibits growth of hypoxic HR-proficient NSCLC spheroids. A549 and H3122 spheroids were treated for 7 days with vehicle, talazoparib (20 nM), TPZ (1 μM), or talazoparib + TPZ. Culture medium was refreshed every two days, and spheroid growth was monitored using a Celigo cytometer throughout the experiment. **A**, **B** Representative bright-field images of A549 (**A**) and H3122 (**B**) spheroids during the 7-day treatment. Analysis of spheroid growth curves for A549 and H3122 revealed that TPZ inhibited spheroid growth, and this effect was further enhanced by the combination with talazoparib. Scale bars: 500 μm. Data are presented as means ± SD (*n* = 3). Statistical significance was determined by one-way ANOVA with Dunnett’s multiple comparisons.

To evaluate the efficacy of talazoparib and TPZ in a hypoxic tumour-like microenvironment, we established 3D spheroid models using a panel of HR-proficient NSCLC cell lines. Among four NSCLC cell lines (Calu-3, H3122, A549, CORL105) we tested, only A549 and H3122 formed compact spheroids with stable growth over 7 days (Fig. 5A, B, Fig. S4A). Immunohistochemistry (IHC) staining for CA9, a hypoxia marker, confirmed the presence of extensive hypoxia in both A549 and H3122 spheroids (Fig. S4B). A549 and H3122 spheroids were treated for 7 days with vehicle, talazoparib (20 nM), TPZ (1 μM) or the combination, with medium refreshed every 2 days. Spheroid growth curves showed that talazoparib alone did not significantly inhibit the growth of A549 and H3122 spheroids, whereas TPZ alone did, indicating the potential effect from hypoxia. The combination further inhibited growth of spheroids compared with single agents by day 7. These findings support our 2D culture results and demonstrate that TPZ and talazoparib enhance cytotoxicity in hypoxic HR-proficient NSCLC tumour cells.

### The combination of talazoparib and TPZ inhibits the growth of hypoxic HR-proficient NSCLC tumours

Given that tumour hypoxia in vivo is highly heterogeneous and influenced by the tumour microenvironment, we performed in vivo studies. To assess whether the combination of talazoparib and TPZ enhances therapeutic efficacy in hypoxic HR-proficient tumours, we established a panel of human NSCLC subcutaneous xenografts in athymic nude mice and quantified their hypoxia levels using CA9 IHC staining. Hypoxic fractions across the xenograft models ranged from 1.7% to 18.7% (Fig. S5A, B). A549 and CORL105 xenografts exhibited the highest hypoxic fractions (A549: 18.2 ± 3.1%; CORL105: 18.7 ± 2.8%), whereas Calu-3 xenografts showed the lowest hypoxic fraction (Calu-3: 1.7 ± 0.6%). This finding is consistent with our previous report, showing minimal CA9 positivity in Calu-3 xenografts [23]. Interestingly, in the in vivo setting, H3122 tumours also showed comparatively low levels of hypoxia (H3122: 5.5 ± 1.8%). Based on these findings, A549 and CORL105 xenografts were selected as highly hypoxic tumour models to investigate the impact of hypoxia on tumour response to the combination treatment, while H3122 and Calu-3 xenografts served as low hypoxic tumours.

**Fig. 6:**
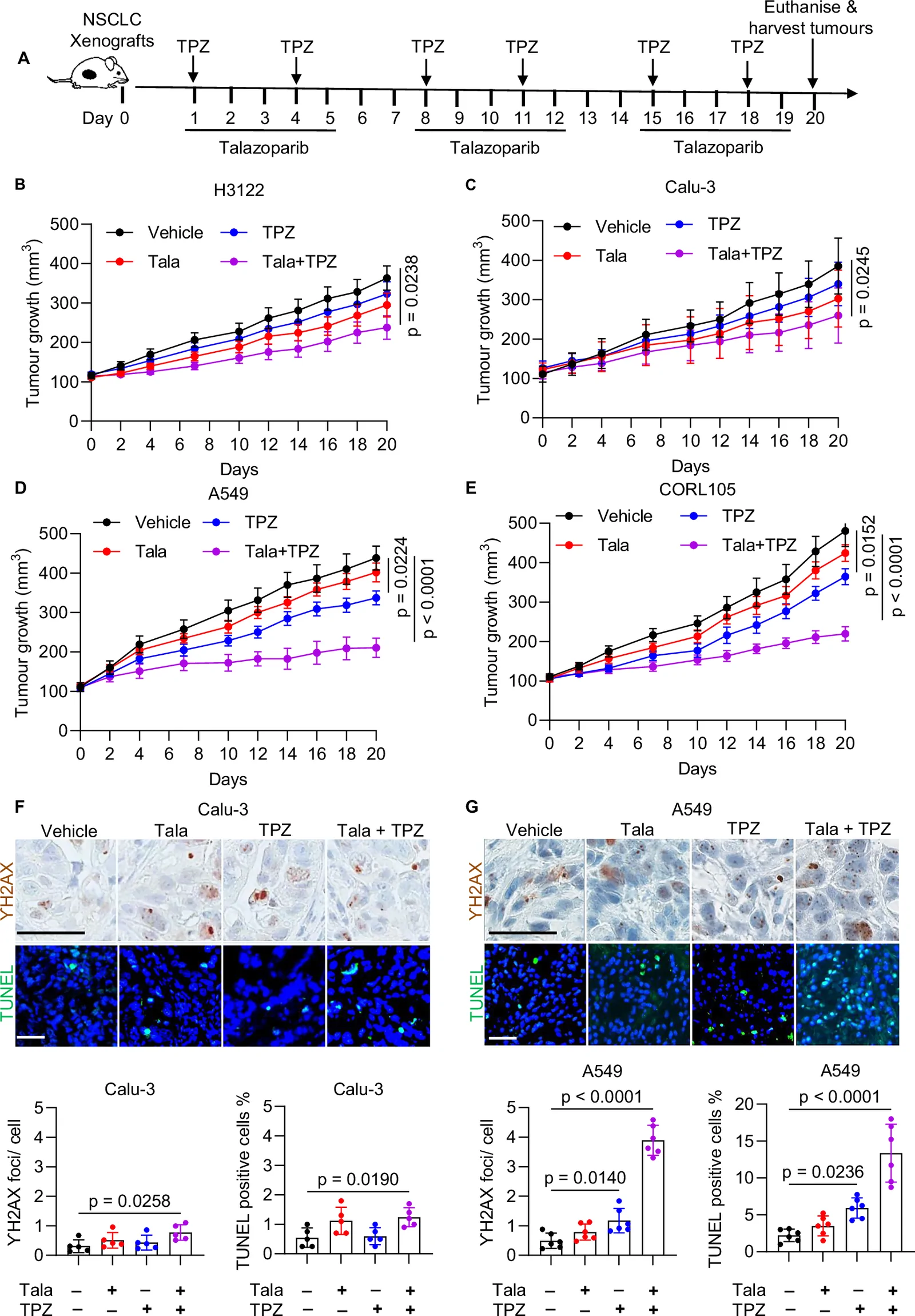
Combination of talazoparib with TPZ inhibits the growth of hypoxic HR-proficient tumours. **A** Schematic diagram of the in vivo experimental design. Nude mice bearing HR-proficient NSCLC subcutaneous xenografts (approximately 100 mm^3^) were treated with vehicle, talazoparib (0.3 mg/kg), TPZ (20 mg/kg,), or the combination on the indicated days (*n* = 5–6/group). **B**–**E** Tumour growth was monitored throughout the treatment period. In the less hypoxic Calu-3 (**B**) and H3122 (**C**) models, tumour growth delay was observed only with the combination treatment. In contrast, in the more hypoxic A549 (**D**) and CORL105 (**E**) tumours, TPZ alone reduced tumour growth, and this effect was further enhanced by the combination with talazoparib. **F**, **G** Mice were euthanised and tumours were collected on day 20 for assessment of DNA damage by γH2AX IHC and apoptosis by TUNEL staining. Representative images of γH2AX and TUNEL staining in Calu-3 (**F**) and A549 (**G**) tumours. Scale bars: 50 μm. Data are presented as means ± SEM (*n* = 5–6). Statistical significance was determined by one-way ANOVA with Dunnett’s multiple comparisons.

Tumour-bearing mice received vehicle, talazoparib (0.3 mg/kg), TPZ (20 mg/kg), or the combination on the indicated schedule following previously established treatment protocols (Fig. 6A) [16, 24]. Tumour size and body weight were monitored regularly throughout the study. As observed in vitro, neither monotherapy alone significantly affected tumour growth in the low hypoxia Calu-3 and H3122 models, whereas the combination treatment led to a reduction in tumour growth (Fig. 6B, C). In contrast, in highly hypoxic A549 and CORL105 models, which also grow more rapidly, TPZ alone reduced tumour growth and the combination with talazoparib induced greater inhibition than either agent alone (Fig. 6D, E). These in vivo findings support our in vitro data showing that the therapeutic efficacy of the talazoparib and TPZ combination is strongly enhanced in tumours with high hypoxic fractions.

To assess potential normal tissue toxicity of the combination treatment, mouse body weight and histology of major organs were examined. The combination treatment was well-tolerated in mice, with average weight loss under 8% (Fig. S6A–D). Histological analysis of A549 tumour-bearing mice revealed no treatment-related morphological abnormalities in normal tissues (Fig. S6E), indicating a lack of systemic toxicity.

### The combination of talazoparib and TPZ increases DNA damage and apoptosis in hypoxic HR-proficient tumours in vivo

We next examined whether antitumour effects of the talazoparib and TPZ combination correlate with changes in DNA damage and apoptotic cell death. Tumour samples from Calu-3 (less hypoxic) and A549 (more hypoxic) models were collected following the efficacy study and analysed for γH2AX and apoptosis (TUNEL assay). In less hypoxic Calu-3 tumours, neither monotherapies significantly altered γH2AX foci (*p* > 0.05 *vs* vehicle) or TUNEL-positive cells (*p* > 0.05 *vs* vehicle) (Fig. 6F). In contrast, in the highly hypoxic A549 tumours, TPZ alone significantly elevated both γH2AX foci and TUNEL-positive cells (Fig. 6G). Notably, the combination of talazoparib and TPZ further increased γH2AX foci and TUNEL-positive cells (Fig. 6F, G), with a particularly pronounced effect in hypoxic A549 tumours, indicating that the combination induces robust DNA damage and apoptosis in this hypoxic model. Together, these data highlight hypoxia-dependent accumulation of DNA damage and apoptosis as key mechanisms underlying the antitumour efficacy of talazoparib combined with TPZ in hypoxic HR-proficient tumours.

## Discussion

In this study, we demonstrate that HR-proficient NSCLC cells can be potently sensitised to talazoparib through the hypoxia-selective cytotoxin TPZ, with the strongest effects under hypoxic conditions. Mechanistically, the TPZ–talazoparib combination markedly increased DNA damage, replication stress, and ROS production with substantially greater effects in hypoxia. Following drug withdrawal, most DNA damage and replication stress were rapidly resolved under normoxia. In contrast, hypoxic cells retained high levels of γH2AX and phospho-RPA, indicating persistent DNA lesions and impaired repair. This persistence likely accounts for the greater synergy observed under hypoxia, as TPZ predominantly induces SSBs that are repaired by PARP-dependent BER and SSB repair pathways [25]. Inhibition of PARP by talazoparib prevents efficient repair of these lesions, leading to their conversion into replication stress-associated DSBs, and thereby amplifying cytotoxicity. Considering previous reports that severe or chronic hypoxia suppresses HR repair capacity [9, 14, 15, 26], we suggest that DNA damage induced by the combination treatment remains unrepaired under hypoxia (1% O_2_), promoting persistent DSB accumulation, increased replication stress, and ultimately cell death. Consistent with this, RAD51 analysis indicated altered HR dynamics under hypoxia. Despite reduced RAD51 protein levels, RAD51 foci formation was increased following TPZ and combination treatment, particularly under hypoxic conditions, suggesting that this response may be driven by elevated DNA damage and replication stress rather than fully effective HR repair.

In addition to direct DNA damage, we observed a marked increase in ROS levels following combination treatment. TPZ elevated ROS predominantly under hypoxia, while talazoparib increased ROS under normoxia, consistent with a previous report [22]. Importantly, the combination further augmented ROS under both oxygen conditions. As ROS can generate oxidative DNA lesions and trigger replication stress, these findings suggest that ROS act as a mechanistic amplifier of combination-induced toxicity.

Our in vivo models validate and extend these in vitro findings in a physiologically relevant context. Using a panel of lung cancer xenografts, we identified tumours with varying degrees of hypoxia, highlighting the heterogeneity of tumour oxygenation in vivo. The modest effects of the TPZ–talazoparib combination observed under normoxia in vitro were insufficient to induce substantial cell death. In vivo, however, tumour heterogeneity, including the presence of hypoxic areas, likely amplifies and sustains combination-induced damage, contributing to antitumour activity even in less hypoxic tumours, while the most pronounced therapeutic effects were observed in severely hypoxic tumours. Consistent with this, the TPZ–talazoparib combination significantly inhibited tumour growth and increased DNA damage and apoptosis in highly hypoxic xenografts, whereas responses were more limited in less hypoxic models.

The impact of hypoxia on PARPi sensitivity has been reported previously. Severe hypoxia (≤ 0.5% O_2_) increases PARPi sensitivity, whereas mild hypoxia (2% O_2_) is associated with resistance [8, 14, 16]. In our study, although RAD51 expression was reduced under 1% O₂, PARPi sensitivity remained largely unchanged in HR-proficient NSCLC. These findings likely reflect variations in baseline replication stress and DNA damage across oxygen levels. Under extreme hypoxia (< 0.1% O_2_), cells experience profound replication stress and impaired DNA synthesis, increasing reliance on HR-mediated replication restart [27, 28]. As HR is compromised under these conditions, PARPi further exacerbates replication-associated DNA damage. In contrast, under mild hypoxia (e.g. 2% O_2_), baseline DNA damage is often reduced compared with normoxia (21% O_2_) due to lower oxidative stress, and replication stress is minimal [16]. As a result, cells are less dependent on HR, rendering PARPi less effective. Our experimental conditions (1% O₂) may represent an intermediate state that is insufficient to generate substantial replication stress or robust HR suppression, resulting in the limited effect of PARPi monotherapy.

Although TPZ showed promise in preclinical and early clinical studies, it failed to demonstrate therapeutic benefit in phase III trials, primarily due to inadequate patient stratification for tumour hypoxia levels [29]. Our in vivo data reinforce this lesson: combining TPZ with talazoparib significantly inhibited the growth of highly hypoxic A549 and CORL105 tumours but had minimal effect in less hypoxic Calu-3 and H3122 tumours. This pattern aligns with the oxygen-dependent mechanism of TPZ and underscores the importance of tumour hypoxia as a predictive marker for this combination strategy.

Several randomised phase III trials have shown that PARPi monotherapy at clinically tolerable doses had limited benefit in HR-proficient cancers [10, 30–32]. Unfortunately, previous attempts to enhance PARPi efficacy through combination with DNA-damaging agents have often been hampered by unacceptable normal tissue toxicity [33]. In contrast, our findings indicate that combining PARPi with TPZ enhances antitumour activity in HR-proficient tumours without additional normal tissue toxicity, suggesting a more selective and tolerable therapeutic approach.

Despite the promising effects observed in this study, several limitations should be considered. Tumour hypoxia is inherently heterogeneous and dynamic, both spatially and temporally, which may influence the uniformity and durability of responses to this combination strategy. As such, the efficacy of combining PARP inhibition with hypoxia-activated agents is likely to vary depending on the extent and persistence of hypoxic regions within individual tumours.

In addition, while our study focuses on TPZ as a hypoxia-activated DNA-damaging agent, other hypoxia-targeting strategies may also be considered. These include hypoxia-activated prodrugs such as evofosfamide, which preferentially undergo activation under more severe hypoxic conditions [34–36], as well as clinically established hypoxia modifiers such as nimorazole, a nitroimidazole-based radiosensitiser widely used in the treatment of hypoxic tumours [37]. Exploration of these approaches may further expand the therapeutic potential of PARP inhibitor-based combination strategies in HR-proficient cancers.

In summary, our study demonstrates that hypoxia potentiates the synergistic antitumour effects of TPZ and PARPi in HR-proficient cancer cells by exacerbating DNA damage, ROS production, and impairing HR-mediated repair. These findings challenge the conventional view of hypoxia as solely a barrier to effective cancer therapy and instead highlight its potential as a therapeutic vulnerability in targeting HR-proficient tumours. This study provides preclinical evidence supporting the combination of hypoxia-activated DNA damaging agents with talazoparib as a strategy to treat hypoxic and HR-proficient cancers, including NSCLC.

## Materials and methods

### Cell culture and drugs

Human lung carcinoma cell lines A549, H3122, CORL105, Calu-3, Calu-6, HCC827, H2087, H1395, and H1975 were purchased from the American Type Culture Collection (ATCC, Rockville, MD, USA). DLD1 parental and BRCA2-deleted DLD1 cells were obtained from Dr. Anderson Ryan’s laboratory [21]. All cell lines were cultured in advanced DMEM/F12 medium (GIBCO), supplemented with 5% FBS, 2 mM glutamax, and 50 µg/ml penicillin/streptomycin. Cells were cultured under normoxic conditions (21% O_2_ and 5% CO_2_) in a regular incubator, or hypoxic conditions (1% O_2_ and 5% CO_2_) in a sealed hypoxic workstation (Baker Ruskinn Technology Ltd, UK).

The following inhibitors and drugs were used: talazoparib (in vitro: 10 mM in DMSO, MedChem Express, Cat: HY-16106-1 ml, in vivo: MedChem Express, Cat: HY-16106-10 mg) and TPZ (in vitro: 10 mM in DMSO, Stratech Scientific, Cat: B3399, in vivo: Sigma, Cat: SML0552).

### Cell viability assay and combenefit analysis

The cytotoxic effects of TPZ and talazoparib in cancer cells were assessed using a CCK-8 assay kit (Stratech Scientific Ltd., Cat: E-CK-A362-ELA). NSCLC cells were seeded in 96-well plates and treated with various concentrations of TPZ (0.1–50 μM), talazoparib (2–100 nM) or their combination for 4 days under normoxic (21% O_2_) or hypoxic (1% O_2_) conditions. Following treatment, 10 μl of CCK-8 reagent was added to each well and incubated at 37 °C for 2 h. Absorbance was measured at 450 nm using a microplate reader. The combination effects of TPZ and talazoparib were quantified and visualised using the Combenefit software, with synergy scores calculated according to the Highest Single Agent (HSA) additivity model [38].

### Comet assays

Cells were treated for 24 h under normoxic or hypoxic conditions with one of the following: DMSO, talazoparib (20 nM), TPZ (1 μM), or a combination of talazoparib and TPZ, and then harvested for alkaline [39] and neutral comet assays [40], performed as previously described with modifications. Briefly, cells were mixed with 1% low melting point agarose (37 °C) and loaded onto microscope slides pre-coated with 1% normal melting point agarose. After the agarose solidified, the slides were immersed in alkaline or neutral lysis buffer (4 °C) for 2 h to lyse cells. DNA was then electrophoresed at 25 V for 25 min. For neutral comet assays, slides were stained directly with SYBR Gold (Invitrogen) for 30 min. For alkaline comet assays, slides were neutralised prior to SYBR Gold staining. Over 100 randomly selected cells per sample were imaged using Nikon eclipse 55i fluorescent microscope (Nikon UK Ltd). DNA damage was quantified using the Olive tail moment (the distance between the centres of the head and tail x the rate of DNA in the tail). Quantification was performed using the OpenComet plugin in Fiji software.

### Intracellular ROS assay

Cells were treated for 24 h under normoxic or hypoxic conditions with one of the following: vehicle, talazoparib (20 nM), TPZ (1 μM), or talazoparib + TPZ. Following treatment, cells were incubated with medium containing 5 μM CellRox Green reagent (Invitrogen, Cat: C10444) at 37 °C in the dark for 30 min. Cells were then washed with PBST, fixed with 4% PFA for 10 min, and resuspended in PBS for flow cytometry. Fluorescent signals were acquired using a CytoFLEX flow cytometer (Beckman Coulter) and data were analysed using FlowJo software (BD Biosciences).

### γH2AX/ phospho-RPA and RAD51 immunofluorescence imaging

Prior to fixation and staining, cells were grown on glass coverslips placed in 6-well plates overnight. For HR proficiency experiments, cells were irradiated with 6 Gy in a ^137^Cs sealed source irradiator (GSR D1, Gamma-Service Medical, Leipzig, Germany) and incubated for 4 h prior to fixation for RAD51 foci analysis. For DNA damage response experiments, cells were treated with DMSO, talazoparib (20 nM), TPZ (1 μM), or talazoparib + TPZ under normoxic or hypoxic conditions for 48 h. After treatment, drug-containing medium was replaced with fresh drug-free medium under normoxia or hypoxia for cell recovery. Cells were collected either immediately (0 h drug withdrawal) or after 24 h drug withdrawal for γH2AX/p-RPA immunofluorescence staining to assess DNA damage and replication stress resolution. RAD51 immunofluorescence staining was performed at 0 h post-drug withdrawal to evaluate HR recruitment at the time of maximal DNA damage. Cells were fixed with 4% paraformaldehyde (PFA) for 15 min and permeabilised with 0.25% triton X-100 in PBS for 10 min. Nonspecific binding was blocked by incubating cells in 5% goat serum/ 2% BSA/ 0.25% Triton X-100/PBS for 1 h at room temperature. Cells were then incubated overnight at 4 °C with primary antibodies: mouse anti-γH2AX antibody (1:500, Millipore, Cat: 05-636), rabbit anti-phospho-RPA32 (S33) antibody (1:500, ThermoFisher, Cat: A300-246A), and rabbit anti-RAD51 antibody (1:500, Abcam, Cat: ab63801). After washing, cells were incubated for 1 h at room temperature with Alexa 488-labelled goat anti-mouse antibody (1:400, Invitrogen, Cat: A11001) and/or Alexa 555-labeled goat anti-rabbit antibody (1:400, Invitrogen, Cat: A21428). Coverslips were mounted using fluorescence mounting medium with DAPI (Abcam, Cat: ab104139). Images were acquired using a Nikon Eclipse 55i fluorescent microscope (Nikon UK Ltd) or an EVOS M5000. DNA damage and replication stress were quantified by counting cells with > 10 γH2AX foci, p-RPA foci, or RAD51 foci, respectively. Approximately 200 cells per sample were analysed using Fiji image analysis software.

### Western blotting

A549 and H3122 cells were seeded in 10 cm plates overnight prior to incubation for 48 h under normoxic or hypoxic conditions. After the incubation period, cells were washed with PBS, detached from the plate and briefly centrifuged before being lysed using UTB lysis buffer (75 mM Tris, 9 M urea and 0.1% (v/v) 2-mercaptoethanol pH 7.5). Protein lysates were briefly sonicated and centrifuged at 8,500 g for 10 minutes at 4 °C and supernatant was used. Protein concentration was determined using the NanoDrop One/OneC Microvolume UV-Vis Spectrophotometer, with absorbance measured at 280 nm. 50 μg of protein were prepared using Bolt LDS Sample Buffer (B0007, Thermo Fisher) and Bolt Sample Reducing Agent (B0009, Thermo Fisher), and denatured at 95 °C. Samples were loaded on a Bolt 4–12% Bis-Tris Plus (NW04120BOX, Invitrogen) using MES SDS Running Buffer (B0002, Invitrogen), and a Precision Plus Protein Kaleidoscope (1610375, Bio-Rad) was included as a molecular weight marker. Samples were run at 150 V for 45 min. The Trans-Blot Turbo Transfer buffer (10026938, Bio-Rad) and Trans Blot Turbo Transfer system were used to perform semi-dry transfer using Immun-Blot PVDF membranes. After the transfer, membranes were blocked in a 5% BSA solution in TBS with 1% Tween-20 (TBST) for an hour prior to incubation with anti-RAD51 antibody (1:1000, Abcam, Cat: ab63801) overnight at 4 °C. Membranes were washed with TBST and incubated with anti-rabbit or β-actin HRP (sc47778, Santa Cruz, 1:5000) for 1 h. Clarity Western ECL Substrate (1705060, Bio-Rad) was used for developing the membranes and the protein bands visualized in a Bio-Rad ChemiDoc system.

### Spheroid experiments

A549 and H3122 cells were seeded at 200 cells/well into BioFloat ultra-low attachment 96-well plates (Swift Analytical, Cat: SWF202003). Spheroids formed within 72 h after seeding. Spheroids were then treated with vehicle, talazoparib (20 nM), TPZ (1 μM), or talazoparib + TPZ, respectively. Drug-containing medium was refreshed every two days. Spheroids were maintained in the presence of drugs for 7 days, and their growth was monitored by imaging every two days using a Celigo cytometer (Nexcelom Bioscience).

### Subcutaneous xenografts

All animal experiments were conducted under the project licence (PP4558762) issued by the Home Office. NSCLC subcutaneous xenografts were established as previously described [23]. Briefly, 6-8 weeks old female athymic nude mice (Charles River, Wolverhampton, UK) were anaesthetised with 2% isoflurane, and 5 × 10^6^ NSCLC cells (A549, H3122, CORL105, Calu-3, Calu-6, HCC827, H2087, H1395, or H1975) suspended in 50% Matrigel (BD Biosciences) were subcutaneously injected into the flank. Mouse body weights and tumour volumes were measured 2-3 times per week. Tumour volume = 1/2 x length x width x depth.

### Treatment schedules

When tumour volumes reached around 100–150 mm^3^, mice bearing A549, H3122, CORL105, Calu-3, Calu-6, HCC827, H2087, H1395, or H1975 xenografts were euthanised, and tumours were harvested for CA9 immunohistochemistry staining (*n* = 3/group).

For the treatment, when tumours reached around 100-150 mm^3^, mice with A549, H3122, CORL105, or Calu-3 xenografts were randomised into four groups (*n* = 5–6/group): (i) vehicle group: oral gavage of 10% dimethylacetamide/ 6% solutol HS/84% PBS once daily for 3 weeks (5 days/week). (ii) Talazoparib group: oral gavage of talazoparib (0.3 mg/kg) once daily for 3 weeks (5 days/week). (iii) TPZ group: intraperitoneal (i.p.) injection of TPZ (20 mg/kg) twice a week on days 1, 4, 8, 11, 15, and 18. (iv) Combination group: oral gavage of talazoparib (0.3 mg/kg) once daily for 3 weeks (5 days/week), with i.p. injection of TPZ (20 mg/kg) 3 h after talazoparib dosing on day 1, 4, 8, 11, 15, and 18. Tumour sizes were measured with a calliper 2–3 times a week. All mice were euthanised on day 20. Tumours were harvested for immunohistochemistry, and normal tissues (heart, lung, spleen, kidney, liver, and small intestine) were harvested for histological examination.

### Histology and immunohistochemistry

Formalin-fixed, paraffin-embedded normal tissue and xenograft tumour sections (4 μm) were deparaffinised in CitroClear and rehydrated through a graded ethanol series. Normal tissue sections were processed for histological examination. Briefly, sections were stained with Harris’ Haematoxylin (VWR International Ltd, Cat: 517-28-2) for 5 min, and rinsed in tap water. Sections were then differentiated in acid alcohol for 10 s, followed by bluing in Scott’s tap water and 0.5% lithium carbonate. Sections were dehydrated through ethanol, counterstained with alcoholic Eosin (Merck Life Science, Cat: HT110116) for 2 min. Sections were dehydrated in absolute ethanol and cleared in xylene.

Tumour sections were processed for immunohistochemistry staining as described previously [9]. Briefly, tumour sections underwent heat-induced antigen retrieval in citrate buffer (Sigma, Cat: C9999), and were blocked with Dual Endogenous Enzyme Block (Agilent, Cat: S200389-2) for 1 h at room temperature. Sections were incubated overnight at 4 °C with primary antibodies against CA9 (1: 500, Invitrogen, Cat: PA5-111410) and γH2AX (1:1000, Millipore, Cat: 05-636). After washing, sections were incubated with either EnVision - HRP mouse system (Agilent, Cat: K400111-2) or EnVision - HRP rabbit system (Agilent, Cat: K400311-2) for 30 min at room temperature. Signal was developed with DAB substrate (Agilent, Cat: K346889-2), followed by haematoxylin counterstaining. Stained slides were scanned using the Aperio CS scanner (Aperio Technologies) and analysed using ImageScope analysis software (Aperio Technologies). γH2AX foci were manually counted in 200 nuclei per tumour sample.

Apoptosis in tumour sections was assessed using the DeadEnd Fluoromeytric TUNEL system (Promega, Cat: G3250) according to the manufacturer’s instructions. Images were captured using a Nikon Eclipse 55i fluorescent microscope (Nikon UK Ltd), and the percentage of TUNEL-positive cells was quantified from 200 nuclei per sample using Fiji software.

### Statistical analysis

Statistical analyses were performed using GraphPad Prism 10. Comparisons between two groups were conducted using Student’s *t*-test. Comparisons among more than two groups were performed using one-way ANOVA followed by Dunnett’s multiple-comparisons test, or two-way ANOVA followed by Tukey’s or Sidak’s multiple-comparisons test, as appropriate. Statistical significance was defined as *p* < 0.05.

## Supplementary information

Supplementary Figures

Uncropped Western Blot data

## Data Availability

All data generated or analysed during this study are included in this published article and its supplementary information files. Additional data are available from the corresponding author upon reasonable request.

## References

[1] Eustermann S, Wu WF, Langelier MF, Yang JC, Easton LE, Riccio AA, et al. Structural basis of detection and signaling of DNA single-strand breaks by human PARP-1. Mol Cell. 2015;60:742–54. 26626479 10.1016/j.molcel.2015.10.032 PMC4678113

[2] Audebert M, Salles B, Calsou P. Involvement of poly(ADP-ribose) polymerase-1 and XRCC1/DNA ligase III in an alternative route for DNA double-strand breaks rejoining. J Biol Chem. 2004;279:55117–26. 15498778 10.1074/jbc.M404524200

[3] Lord CJ, Ashworth A. PARP inhibitors: synthetic lethality in the clinic. Science. 2017;355:1152–8. 28302823 10.1126/science.aam7344 PMC6175050

[4] Murai J, Huang SY, Das BB, Renaud A, Zhang Y, Doroshow JH, et al. Trapping of PARP1 and PARP2 by Clinical PARP Inhibitors. Cancer Res. 2012;72:5588–99. 23118055 10.1158/0008-5472.CAN-12-2753 PMC3528345

[5] Farmer H, McCabe N, Lord CJ, Tutt AN, Johnson DA, Richardson TB, et al. Targeting the DNA repair defect in BRCA mutant cells as a therapeutic strategy. Nature. 2005;434:917–21. 15829967 10.1038/nature03445

[6] Bryant HE, Schultz N, Thomas HD, Parker KM, Flower D, Lopez E, et al. Specific killing of BRCA2-deficient tumours with inhibitors of poly(ADP-ribose) polymerase. Nature. 2005;434:913–7. 15829966 10.1038/nature03443

[7] Dias MP, Moser SC, Ganesan S, Jonkers J. Understanding and overcoming resistance to PARP inhibitors in cancer therapy. Nat Rev Clin Oncol. 2021;18:773–91. 34285417 10.1038/s41571-021-00532-x

[8] Chan N, Pires IM, Bencokova Z, Coackley C, Luoto KR, Bhogal N, et al. Contextual synthetic lethality of cancer cell kill based on the tumor microenvironment. Cancer Res. 2010;70:8045–54. 20924112 10.1158/0008-5472.CAN-10-2352 PMC2978949

[9] Jiang Y, Verbiest T, Devery AM, Bokobza SM, Weber AM, Leszczynska KB, et al. Hypoxia potentiates the radiation-sensitizing effect of olaparib in human non-small cell lung cancer xenografts by contextual synthetic lethality. Int J Radiat Oncol Biol Phys. 2016;95:772–81. 27020103 10.1016/j.ijrobp.2016.01.035 PMC4856738

[10] Ray-Coquard I, Pautier P, Pignata S, Perol D, Gonzalez-Martin A, Berger R, et al. Olaparib plus Bevacizumab as First-Line Maintenance in Ovarian Cancer. N Engl J Med. 2019;381:2416–28. 31851799 10.1056/NEJMoa1911361

[11] Kaplan AR, Gueble SE, Liu Y, Oeck S, Kim H, Yun Z, et al. Cediranib suppresses homology-directed DNA repair through down-regulation of BRCA1/2 and RAD51. Sci Transl Med. 2019;11:eaav4508. 10.1126/scitranslmed.aav4508 PMC6626544 31092693

[12] Moulder JE, Rockwell S. Hypoxic fractions of solid tumors: experimental techniques, methods of analysis, and a survey of existing data. Int J Radiat Oncol Biol Phys. 1984;10:695–712. 6735758 10.1016/0360-3016(84)90301-8

[13] Vaupel P, Hockel M, Mayer A. Detection and characterization of tumor hypoxia using pO2 histography. Antioxid Redox Signal. 2007;9:1221–35. 17536958 10.1089/ars.2007.1628

[14] Bindra RS, Schaffer PJ, Meng A, Woo J, Maseide K, Roth ME, et al. Down-regulation of Rad51 and decreased homologous recombination in hypoxic cancer cells. Mol Cell Biol. 2004;24:8504–18. 15367671 10.1128/MCB.24.19.8504-8518.2004 PMC516750

[15] Chan N, Koritzinsky M, Zhao H, Bindra R, Glazer PM, Powell S, et al. Chronic hypoxia decreases synthesis of homologous recombination proteins to offset chemoresistance and radioresistance. Cancer Res. 2008;68:605–14. 18199558 10.1158/0008-5472.CAN-07-5472

[16] Mehibel M, Xu Y, Li CG, Moon EJ, Thakkar KN, Diep AN, et al. Eliminating hypoxic tumor cells improves response to PARP inhibitors in homologous recombination-deficient cancer models. J Clin Invest. 2021;131. 10.1172/JCI146256 PMC8266208 34060485

[17] Liu X, Ge Z, Yang F, Contreras A, Lee S, White JB, et al. Identification of biomarkers of response to preoperative talazoparib monotherapy in treatment naive gBRCA+ breast cancers. NPJ Breast Cancer. 2022;8:64. 35538088 10.1038/s41523-022-00427-9 PMC9090765

[18] Moriwaki T, Okamoto S, Sasanuma H, Nagasawa H, Takeda S, Masunaga SI, et al. Cytotoxicity of Tirapazamine (3-Amino-1,2,4-benzotriazine-1,4-dioxide)-Induced DNA Damage in Chicken DT40 Cells. Chem Res Toxicol. 2017;30:699–704. 27943678 10.1021/acs.chemrestox.6b00417

[19] Jones GD, Weinfeld M. Dual action of tirapazamine in the induction of DNA strand breaks. Cancer Res. 1996;56:1584–90. 8603406

[20] Birkelbach M, Ferraiolo N, Gheorghiu L, Pfaffle HN, Daly B, Ebright MI, et al. Detection of impaired homologous recombination repair in NSCLC cells and tissues. J Thorac Oncol. 2013;8:279–86. 23399959 10.1097/JTO.0b013e31827ecf83 PMC3573529

[21] Tacconi EM, Lai X, Folio C, Porru M, Zonderland G, Badie S, et al. BRCA1 and BRCA2 tumor suppressors protect against endogenous acetaldehyde toxicity. EMBO Mol Med. 2017;9:1398–414. 28729482 10.15252/emmm.201607446 PMC5623864

[22] Liu Q, Gheorghiu L, Drumm M, Clayman R, Eidelman A, Wszolek MF, et al. PARP-1 inhibition with or without ionizing radiation confers reactive oxygen species-mediated cytotoxicity preferentially to cancer cells with mutant TP53. Oncogene. 2018;37:2793–805. 29511347 10.1038/s41388-018-0130-6 PMC5970015

[23] Jiang Y, Allen D, Kersemans V, Devery AM, Bokobza SM, Smart S, et al. Acute vascular response to cediranib treatment in human non-small-cell lung cancer xenografts with different tumour stromal architecture. Lung Cancer. 2015;90:191–8. 26323213 10.1016/j.lungcan.2015.08.009 PMC4641245

[24] Froidevaux P, Grilj V, Bailat C, Geyer WR, Bochud F, Vozenin M-C. FLASH irradiation does not induce lipid peroxidation in lipids micelles and liposomes. Radiat Phys Chem. 2023;205:110733.

[25] Abbotts R, Wilson DM 3rd. Coordination of DNA single strand break repair. Free Radic Biol Med. 2017;107:228–44. 27890643 10.1016/j.freeradbiomed.2016.11.039 PMC5443707

[26] Meng AX, Jalali F, Cuddihy A, Chan N, Bindra RS, Glazer PM, et al. Hypoxia down-regulates DNA double strand break repair gene expression in prostate cancer cells. Radiother Oncol. 2005;76:168–76. 16026872 10.1016/j.radonc.2005.06.025

[27] Hammond EM, Denko NC, Dorie MJ, Abraham RT, Giaccia AJ. Hypoxia links ATR and p53 through replication arrest. Mol Cell Biol. 2002;22:1834–43. 11865061 10.1128/MCB.22.6.1834-1843.2002 PMC135616

[28] Pires IM, Bencokova Z, Milani M, Folkes LK, Li JL, Stratford MR, et al. Effects of acute versus chronic hypoxia on DNA damage responses and genomic instability. Cancer Res. 2010;70:925–35. 20103649 10.1158/0008-5472.CAN-09-2715 PMC2923514

[29] Reddy SB, Williamson SK. Tirapazamine: a novel agent targeting hypoxic tumor cells. Expert Opin Investig Drugs. 2009;18:77–87. 19053884 10.1517/13543780802567250

[30] Moore K, Colombo N, Scambia G, Kim BG, Oaknin A, Friedlander M, et al. Maintenance olaparib in patients with newly diagnosed advanced ovarian cancer. N Engl J Med. 2018;379:2495–505. 30345884 10.1056/NEJMoa1810858

[31] Gonzalez-Martin A, Pothuri B, Vergote I, DePont Christensen R, Graybill W, Mirza MR, et al. Niraparib in Patients with Newly Diagnosed Advanced Ovarian Cancer. N Engl J Med. 2019;381:2391–402. 31562799 10.1056/NEJMoa1910962

[32] Coleman RL, Fleming GF, Brady MF, Swisher EM, Steffensen KD, Friedlander M, et al. Veliparib with first-line chemotherapy and as maintenance therapy in ovarian cancer. N Engl J Med. 2019;381:2403–15. 31562800 10.1056/NEJMoa1909707 PMC6941439

[33] Veneris JT, Matulonis UA, Liu JF, Konstantinopoulos PA. Choosing wisely: selecting PARP inhibitor combinations to promote anti-tumor immune responses beyond BRCA mutations. Gynecol Oncol. 2020;156:488–97. 31630846 10.1016/j.ygyno.2019.09.021

[34] Laubach JP, Liu CJ, Raje NS, Yee AJ, Armand P, Schlossman RL, et al. A Phase I/II Study of evofosfamide, a hypoxia-activated prodrug with or without bortezomib in subjects with relapsed/refractory multiple myeloma. Clin Cancer Res. 2019;25:478–86. 30279233 10.1158/1078-0432.CCR-18-1325 PMC6335171

[35] Weiss GJ, Infante JR, Chiorean EG, Borad MJ, Bendell JC, Molina JR, et al. Phase 1 study of the safety, tolerability, and pharmacokinetics of TH-302, a hypoxia-activated prodrug, in patients with advanced solid malignancies. Clin Cancer Res. 2011;17:2997–3004. 21415214 10.1158/1078-0432.CCR-10-3425

[36] Sun JD, Liu Q, Wang J, Ahluwalia D, Ferraro D, Wang Y, et al. Selective tumor hypoxia targeting by hypoxia-activated prodrug TH-302 inhibits tumor growth in preclinical models of cancer. Clin Cancer Res. 2012;18:758–70. 22184053 10.1158/1078-0432.CCR-11-1980

[37] Overgaard J, Hansen HS, Overgaard M, Bastholt L, Berthelsen A, Specht L, et al. A randomized double-blind phase III study of nimorazole as a hypoxic radiosensitizer of primary radiotherapy in supraglottic larynx and pharynx carcinoma. results of the danish head and neck cancer study (DAHANCA) Protocol 5-85. Radiother Oncol. 1998;46:135–46. 9510041 10.1016/s0167-8140(97)00220-x

[38] Di Veroli GY, Fornari C, Wang D, Mollard S, Bramhall JL, Richards FM, et al. Combenefit: an interactive platform for the analysis and visualization of drug combinations. Bioinformatics. 2016;32:2866–8. 27153664 10.1093/bioinformatics/btw230 PMC5018366

[39] Clementi E, Garajova Z, Markkanen E. Measuring DNA damage using the alkaline comet assay in cultured cells. Bio Protoc. 2021;11:e4119. 34541038 10.21769/BioProtoc.4119 PMC8413625

[40] Nickson CM, Moori P, Carter RJ, Rubbi CP, Parsons JL. Misregulation of DNA damage repair pathways in HPV-positive head and neck squamous cell carcinoma contributes to cellular radiosensitivity. Oncotarget. 2017;8:29963–75. 28415784 10.18632/oncotarget.16265 PMC5444717

