# Supplementary material for: Tirapazamine sensitises hypoxic homologous recombination-proficient NSCLC to PARP inhibition
Source: Cell Death Discov. 2026 Jul 2;12:381. doi: 10.1038/s41420-026-03233-5 (PMC13598068; doi:10.1038/s41420-026-03233-5)
Supplement: Supplementary file 1 [file 41420_2026_3233_MOESM1_ESM.pdf]

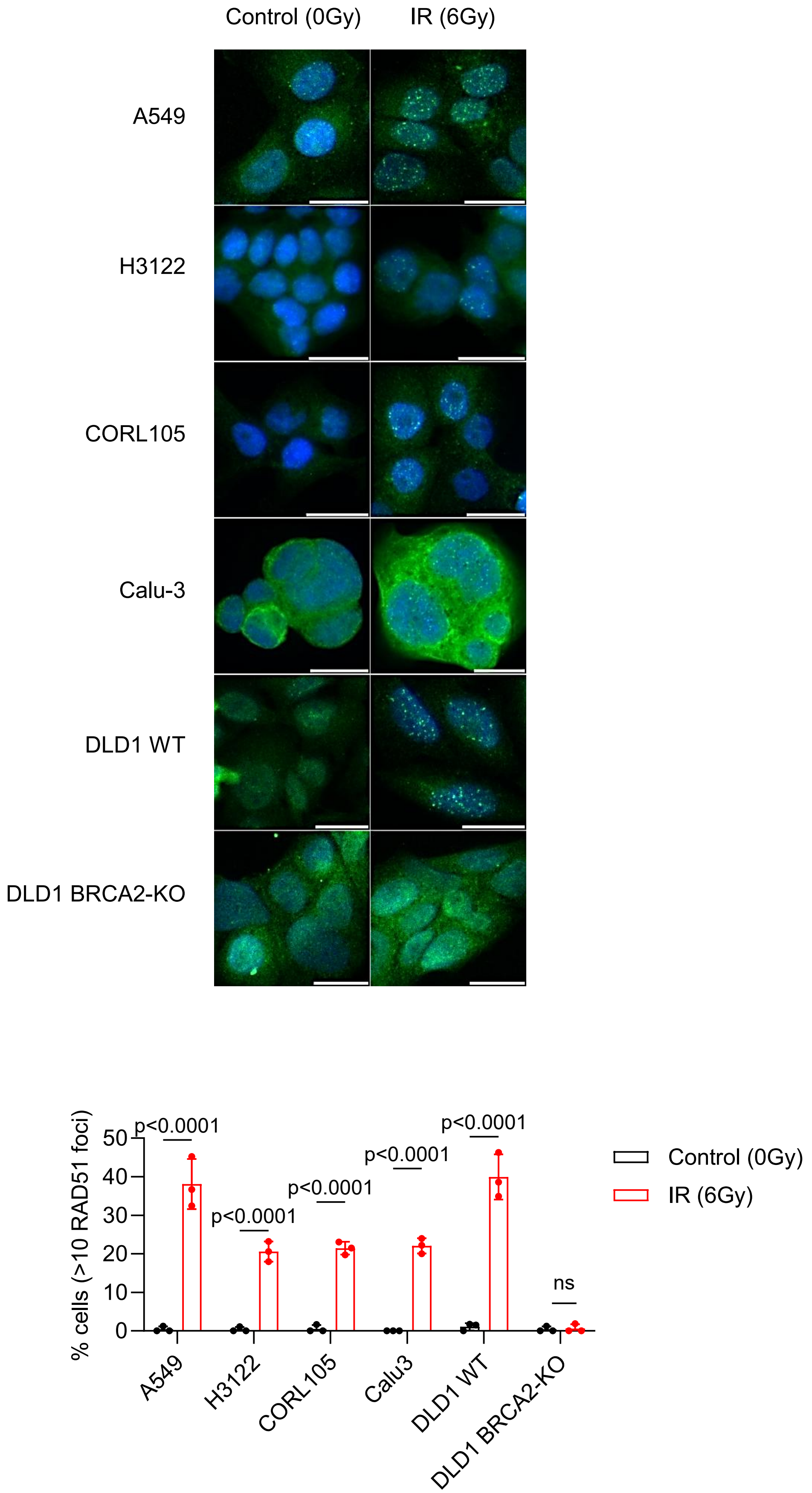

Supplementary Fig. 1 Immunofluorescence analysis of RAD51 foci formation following ionizing radiation. NSCLC cell lines (A549, H3122, CORL105, and Calu-3) were irradiated with 6 Gy and stained for RAD51 (green) and DAPI (blue) 4 hours post-irradiation. All cell lines showed robust induction of RAD51 foci, consistent with functional homologous recombination (HR). DLD1 cells with and without BRCA2 knockout were included as HR-proficient and HR-deficient controls, respectively Scale bars: 20 µm. n = 3, Two-way ANOVA with Sidak's multiple comparisons

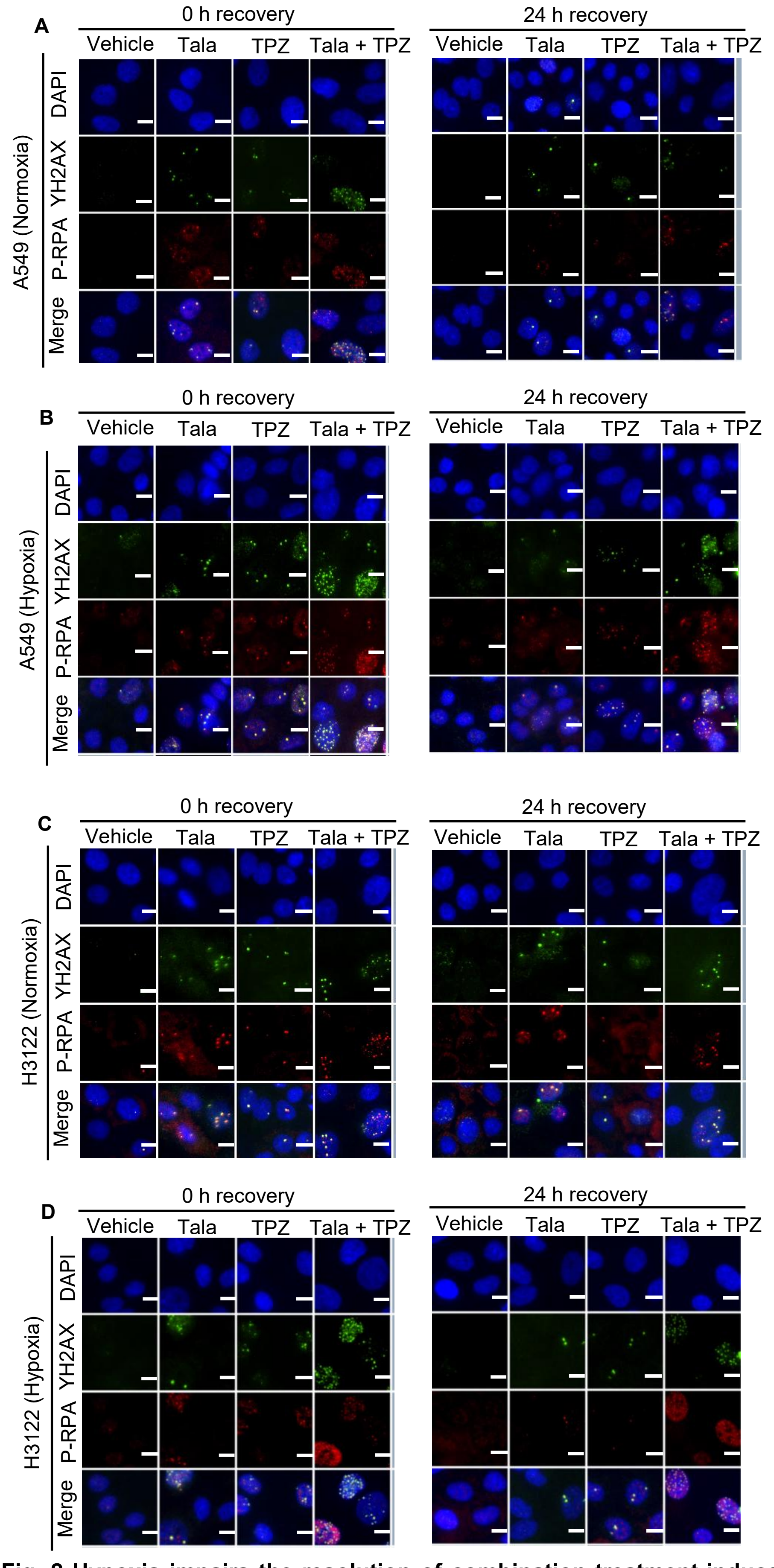

Supplementary Fig. 2 Hypoxia impairs the resolution of combination treatment-induced DNA damage and replication stress. A549 and H3122 cells were treated for 48 h under normoxia or hypoxia with vehicle, talazoparib (20 nM), TPZ (1 μM), or talazoparib + TPZ. Following treatment, drug-containing medium was replaced by drug-free medium under normoxia or hypoxia for cell recovery. Cells were harvested at 0 or 24 h post-drug withdrawal for immunofluorescence staining. (A-D) Representative immunofluorescence staining images of γH2AX (green)/ p-RPA (red)/ DAPI (blue) in: (A) A549 cells under normoxia, (B) A549 cells under hypoxia, (C) H3122 cells under normoxia, and (D) H3122 cells under hypoxia. Scale bars: 10 μm.

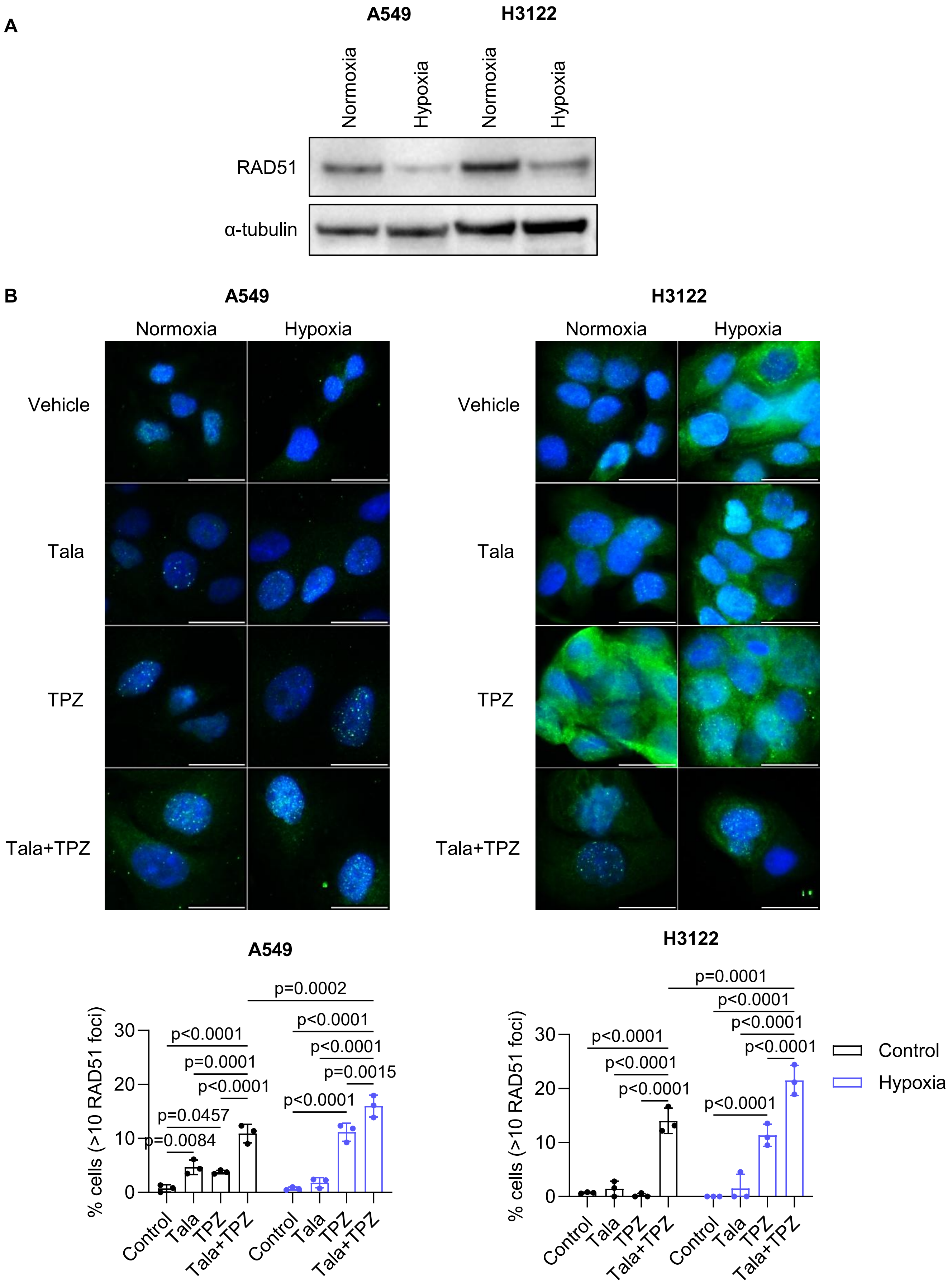

Supplementary Fig. 3 Hypoxia impairs the resolution of combination treatment-induced DNA damage and replication stress. A. RAD51 protein expression is reduced in A549 and H3122 cells following 48 h of exposure to 1% hypoxia. B. A549 and H3122 cells were treated for 48 h under normoxia or hypoxia with talazoparib (20 nM), TPZ (1 μM), or both. Cells were fixed immediately after treatment and stained for RAD51 (green) and DAPI (blue). RAD51 foci (>10/cell) were quantified. Scale bars: 20 μm. n = 3, Two-way ANOVA with Tukey's multiple comparisons

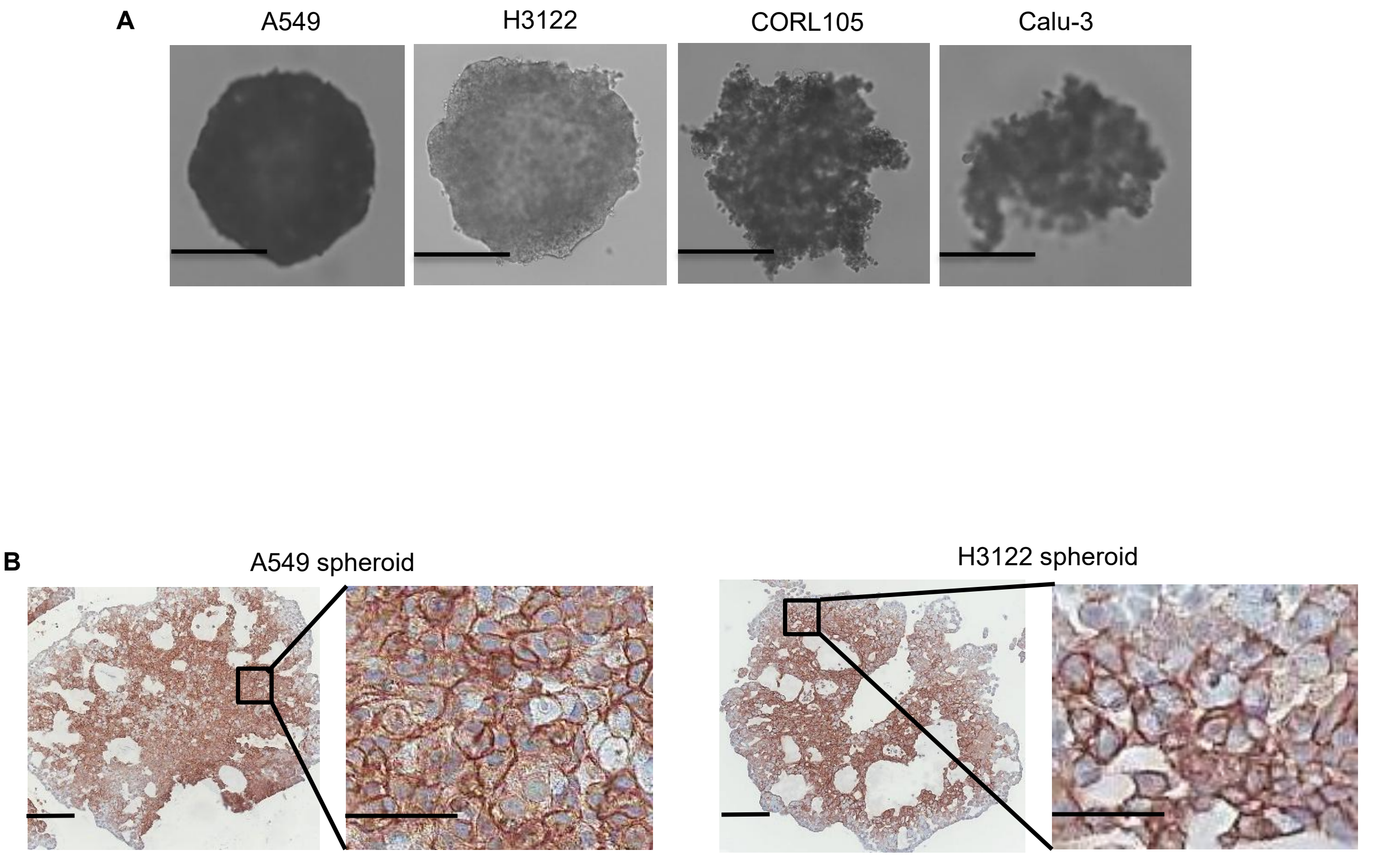

Supplementary Fig 4. HR-proficient NSCLC spheroids. NSCLC spheroids were generated in ultra-low attachment 96-well plates. (A) Representative brightfield images of NSCLC spheroids. Scale bars, 500 μm. (B) Representative CA9 IHC images (brown) in A549 and H3122 spheroids. Scale bars: 200 μm. Inset: 50 μm.

A

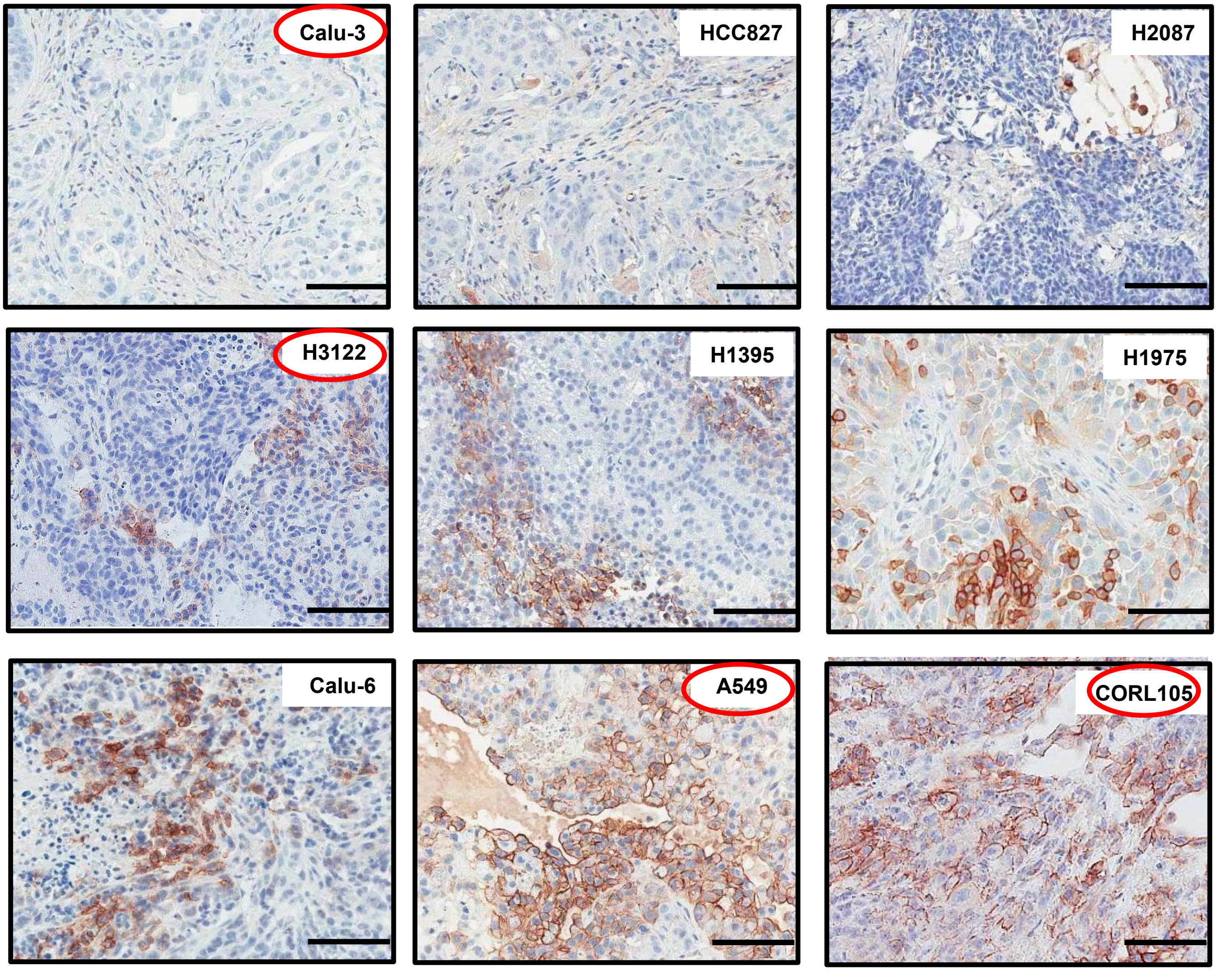

B

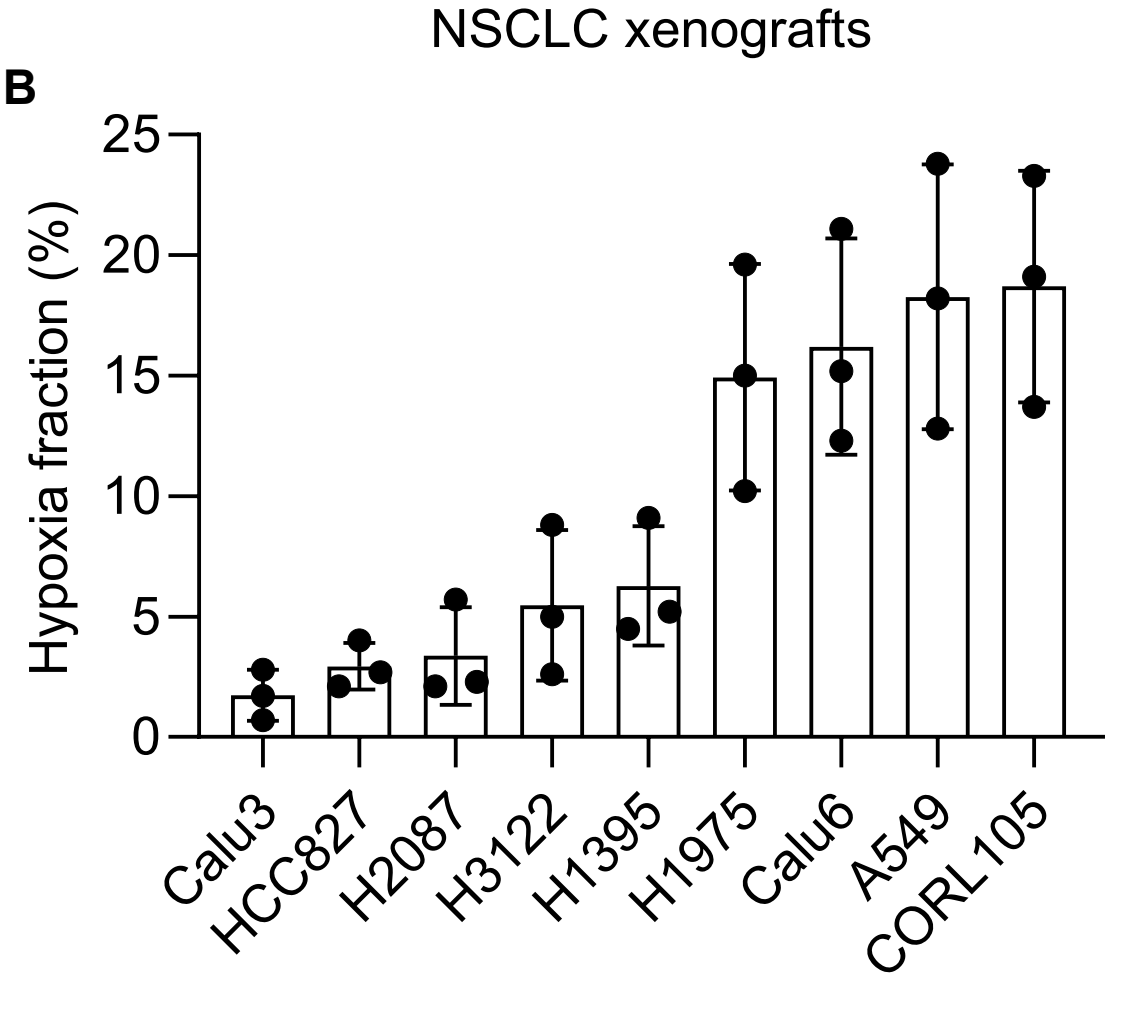

Supplementary Fig 5. NSCLC subcutaneous xenografts show diverse hypoxia levels. Sections from a panel of NSCLC xenografts were stained for CA9, a hypoxic marker, by IHC. (A) Representative images of CA9 IHC staining (brown). Sale bar: 100 µm. (B) Quantification of CA9-positive fractions in xenograft tumours (n=3/ group). Data are presented as means ± SD.

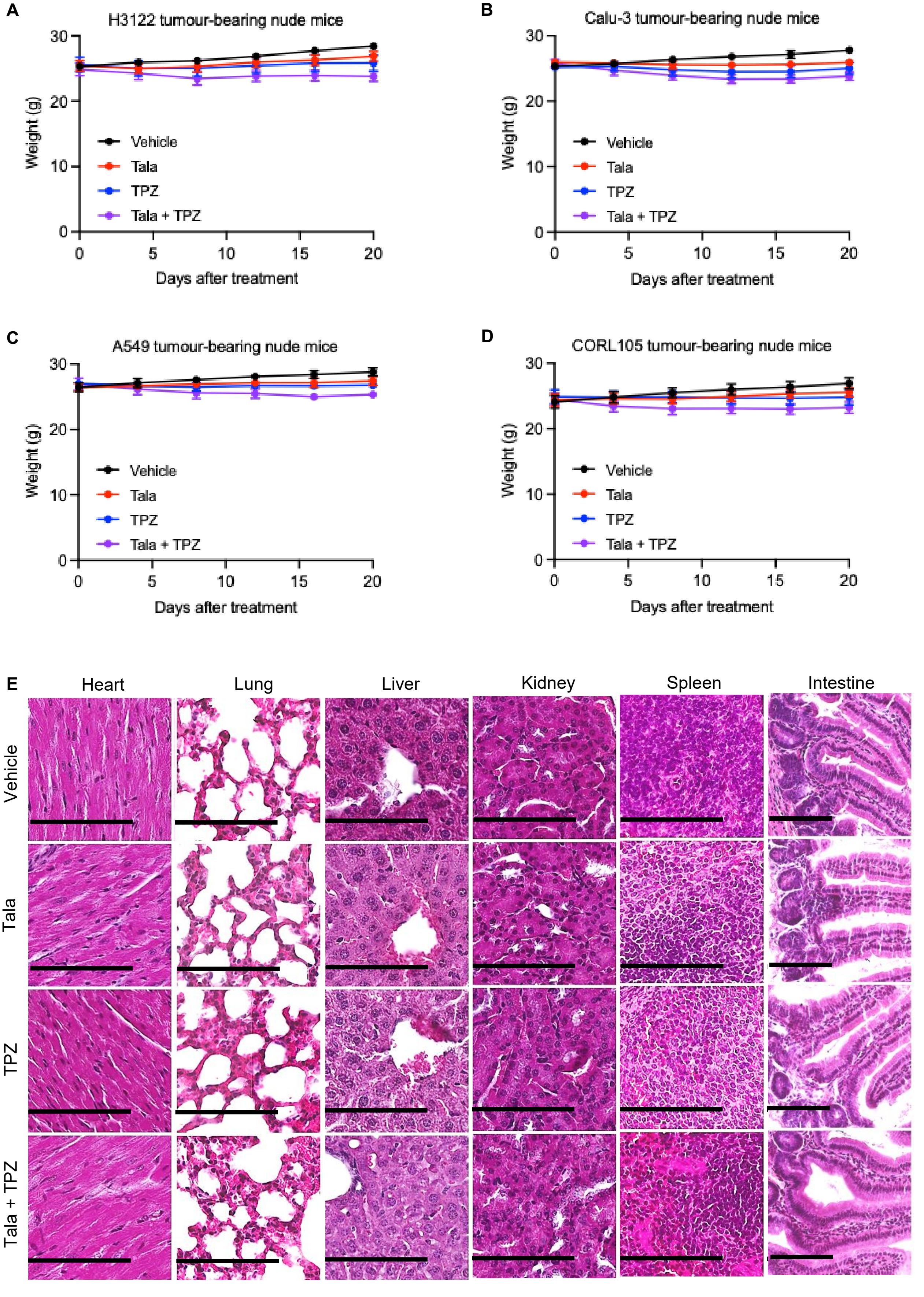

Supplementary Fig 6. Talazoparib combined with TPZ does not cause systematic toxicity. (A-D) Body weights of H3122 (A), Calu-3 (B), A549 (C), and CORL105 (D) tumour-bearing mice were monitored throughout the treatment course. Data are presented as means ± SEM. (E) Heart, lung, liver, kidney, spleen, and intestine were collected at the end of the experiments for histology. Representative HE-stained images of these normal tissues are shown. Scale bars: 100 µm.
