# Supplementary material for: Tirapazamine sensitises hypoxic homologous recombination-proficient NSCLC to PARP inhibition
Source: Cell Death Discov. 2026 Jul 2;12:381. doi: 10.1038/s41420-026-03233-5 (PMC13598068; doi:10.1038/s41420-026-03233-5)

Supplementary Fig. 3A

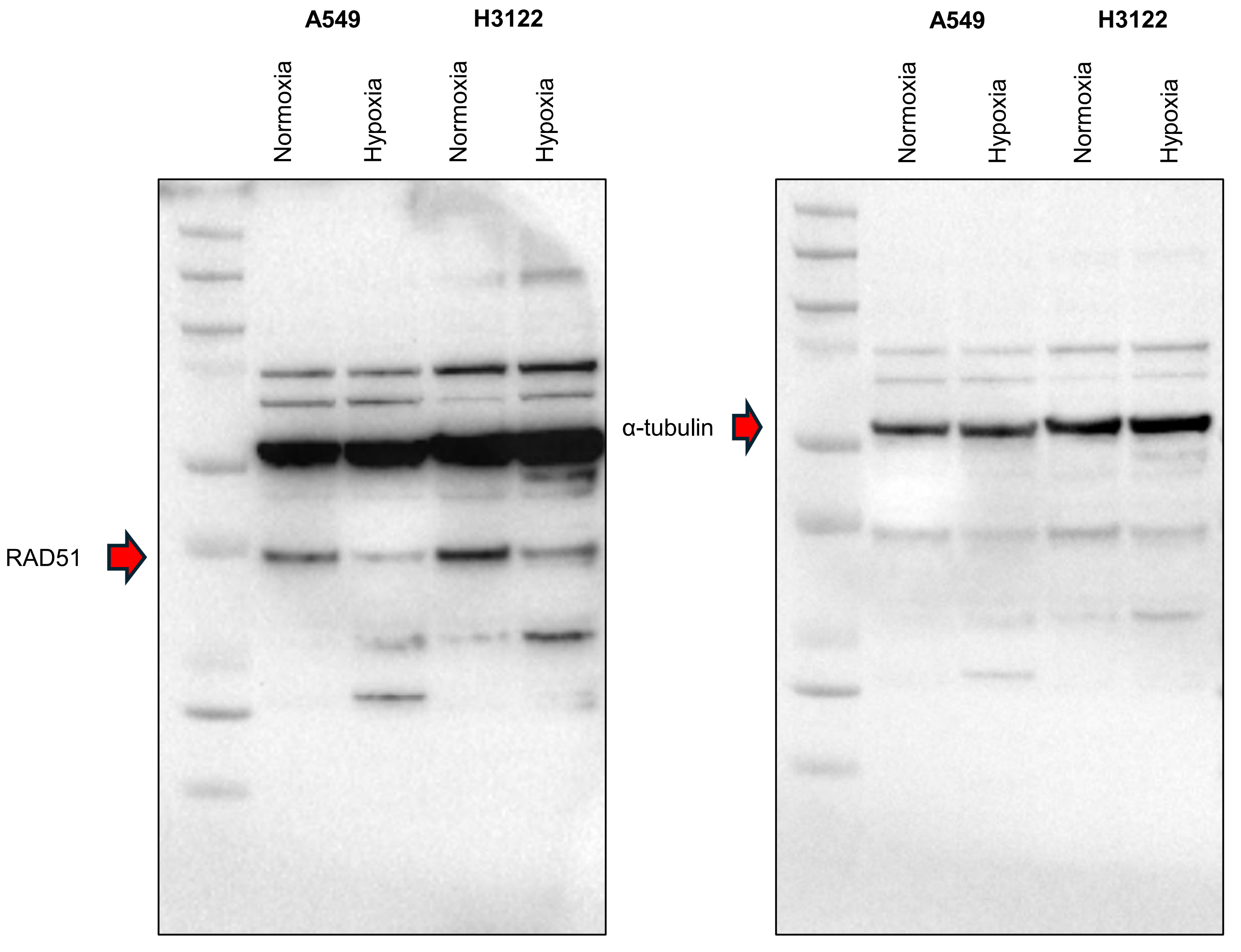

Supplement: Supplementary file 2 [file 41420_2026_3233_MOESM2_ESM.pdf]
